# Recent Developments in the Regulation of Heritable Human Genome Editing

**DOI:** 10.1007/s11673-023-10332-w

**Published:** 2024-04-03

**Authors:** S. Soni

**Affiliations:** School of Law, University of KwaZulu-Natal, Scottsville, Pietermaritzburg, 3209 South Africa

**Keywords:** Genome editing, Health, Bioethics, Autonomy, Law, Regulation

## Abstract

In 2018, the Chinese scientist He Jiankui presented his research at the Second International Summit on Human Genome Editing in Hong Kong. While it was intended that he facilitate a workshop, he was instead called on to present his research in heritable human genome editing, where he made the announcement that he had taken great strides in advancement of his research, to the extent that he had gene-edited human embryos and that this had resulted in the live births of two children. While his research ethic and methodology was interrogated, he insisted that two children, twin girls, had been born healthy and that there was another pregnancy (at the time) where birth of a third gene edited child would be imminent. This announcement generated a ripple effect in the scientific community and exposed the gaps in regulation and absence of law relating to the technology. This resulted in a flurry of activity and conversation around regulation of the technology, which scientists stated was not ready for human trials. This article reviews the Third Summit which was held in London in March 2023 and comments on the latest developments in the regulation of heritable human genome editing.

## Introduction

Human genome editing has the potential to revolution human health. By using gene editing tools such as CrisprCas9 to alter the DNA of an organism, gene editing can make molecular changes to that organism’s DNA to obtain a desired outcome. Research in gene editing has targeted issues in health including the improvement of health outcomes as well as disease control. In this respect we have seen research on the use of gene editing to improve crops to make them hardier and more resistance to environmental stresses. We have also seen research on the use of gene editing to alter the genomes of mosquito to remove their ability to transmit diseases such as malaria. We have also seen research on cattle, with a view to alter their genomes to render them horn-less. However, none of that research has attracted as much human investment and attention as the research on potential applications of gene editing technology to resolving issues in human health.

In 2018, when He Jiankui made his revelation that he had altered the genomes of two children for the purposes of rendering them resistant to HIV infection, the news received mixed reactions. While the scientific community largely condemned his flagrant disregard for the safety of the children involved, the general population remained divided on the issue. On the one hand people were concerned that he had subjected our weakest and most vulnerable to what amounted to human experimentation (the children’s genomes were edited when they were at the embryonic stage of development), but on the other hand many people applauded his attempt at reducing escalating HIV rates. If gene editing has the potential to eradicate diseases such as HIV, cancer, and others, then on what basis should we stop the scientists who are trying to do this?

## Heritable Human Genome Editing

Human genome editing utilizes gene editing technology to make changes to human DNA to achieve a desired change in the genome. We may distinguish between somatic editing, which is gene editing applied to somatic cells. This type of gene editing does not create heritable changes and the changes to the DNA remain with the individual themselves. Apart from establishing the safety and efficacy of the therapy and obtaining the required approvals to bring the therapy to market, this type of gene editing remains largely uncontroversial. On the other hand, heritable genome editing remains concerning as it involves editing the DNA of embryos or gametes, which results in alterations to a genome that is heritable by future generations. Beyond establishing merely safety and efficacy, this type of gene editing is fraught with legal and ethical issues. Since 2018, there has been significant movement in the form of international debate, the drafting of reports and the creation of working groups to consider the ethical and legal issues surrounding heritable genome editing (National Academy of Medicine et al. 2020). This has also stimulated academic debate (Angrist et al. 2020).

## The Third International Summit on Heritable Human Genome Editing

The Third International Summit on Human Genome Editing, which was convened by the U.K. Royal Society, U.K. Academy of Medical Sciences, U.S. National Academies of Sciences and Medicine, and the World Academy of Sciences, aimed to discuss progress, promise, and challenges in research, regulation, and equitable development of human genome editing technologies and therapies. The Summit attracted leading scientists in the field, policymakers and also featured the first patient who had been treated for sickle cell anaemia using gene editing technology. The findings of this third meeting emphasized that remarkable developments had been made in the field of somatic human genome editing, however the high cost of somatic therapies was highlighted as unsustainable and participants called for a global commitment to ensuring equitable access to treatment. The finding in respect of heritable genome editing however was not as positive. Here the finding was that this type of gene editing remains unacceptable as safety and efficacy had not been established, and that the governance frameworks and ethical principles which would apply to responsible use of the technology were not in place (Organizing Committee of the Third International Summit on Human Genome Editing 2023). In this regard, there has been a move in the South African jurisdiction to address these issues.

## The Scope of the Law

In 2020 Baylis et al. undertook a policy survey of 106 countries in order to obtain a clear and accurate understanding of the global policy landscape for human germline and heritable genome editing (Baylis et al. 2020). That study revealed that 96 out of the 106 countries surveyed had policy documents which were relevance to the use of heritable human genome editing. These documents included legislation, regulations, guidelines, codes, and ratification of international treaties. But law takes time to be enacted. The majority of the legal canon which exists today is based on historic law, which has been measured against the “supreme” laws of a country which include Constitutions and other documents which codify the fundamental human rights. The written law which exists today does not describe or mention gene editing because the technology did not exist at the time that the laws were drafted. However, we can try to find some guidance by considering the two broad types of law, which is hard law and soft law. Hard law exists in the form of legislation and statutes, and there are legal consequences for violating these laws (such as fines or imprisonment). Soft law does not carry the force of true law and exists in the form of guidelines as to what may be permissible in a given set of circumstances. For instance, codes of conduct and good practice guidelines for a medical practitioner would fall into this class. While they highlight what is permissible, they themselves are not identified as law by the law-making bodies of a country. They can however indirectly have legal consequences for a person, for instance if a medical practitioner does not follow good practice guidelines, they may lose their licence to practice medicine and that has the legal consequence of the medical practitioners not being able to lawfully practice medicine any longer.

The distinction between these two types of law is important, because we need the hard law to be able to enforce legal principles of good governance that come with a penalty where these laws are contravened. A large part of Jiankui’s ill-reception was based on the disregard for ethics in science, as well as his methodologies employed at various stages in his research. This included the ethical approvals which were obtained, where the experiments were conducted, and how he approached potential participants to participate in the research and permit the editing of their embryo’s genome. However, a significant question at the time was whether Jiankiu had broken any laws when conducting his research. There were no laws in China at the time which prohibited gene editing, and for that reason in the end Jiankui was charged with practicing medicine without the required licence, which was the only charge which the Chinese authorities could lawfully bring against him. The children themselves were gene edited at the embryonic stage, at which point they did not possess any legal recognition as they were not legally recognized persons who would be capable of possessing legal rights, duties, and responsibilities. Because embryos do not possess legal personhood, they do not have rights in the manner which people generally do. However, there is ethical and moral significance which embryos do attract and this can be seen in the law and regulations surrounding research which involve human embryos. The most significant rule in this regard is the “14-day rule” which prohibits research on a human embryo once it reaches the 14^th^ day of development. This rule has been enshrined in the law in the legal systems of many countries, including South Africa, where we see it in a Regulation which supports South Africa’s National Health Act 61 of 2003.

## South Africa’s Suggestions

The Baylis study noted that South Africa did not have “any relevant information” regarding heritable human genome editing. However, research has shown that while gene editing is not expressly mentioned in extant law, there are two statutes which may possibly be applied to the technology in a regulatory fashion (Thaldar and Shozi 2022). The first is the Medicine and Related Substances Act 101 of 1965, where the definition of “medical devices” may be interpreted so as to include gene editing tools. If this were the case, then gene editing tools such as CrisprCas9 would fall under the ambit of the South African Health Products Regulatory Authority. The second possible statute which could be interpreted so as to apply in the context of heritable human genome editing is the National Health Act 61 of 2003. This statute prohibits human cloning as it involves “the manipulation of genetic material in order to achieve the reproduction of a human being.” It has been argued that the legal permissibility of heritable human genome editing may rest on the way in which the words *reproduction of a human being* is interpreted. If interpreted to mean human reproduction, then heritable human genome editing would be unlawful. However, if interpreted narrowly so as to mean the replication of a human being, then the statute would not prohibit gene editing methods that result in heritable changes, and both preclinical and clinical trials for the technology would be subject to ethical clearance requirements by a health research ethics committee in terms of the same statute.

By considering the meaning of the provisions of these two statutes, a set of five ethical principles have been suggested which may be used to draft possible guidelines, as there are currently no guidelines on genome editing in South Africa which could assist the South African Health Products Regulatory Authority or health research ethics committees (Thaldar et al. 2020). In summary, they are the following:

- *Principle I: Given its potential to improve the lives of the people of South Africa, heritable human genome editing should be regulated, not banned.*
- *Principle II: Heritable human genome editing’s clinical applications should be made accessible to the public only if they are proven to be safe and effective.*
- *Principle III: Non-therapeutic heritable human genome editing should be regulated in the same way as heritable human genome editing, with the rider that it should not in any legally relevant sense cause harm.*
- *Principle IV: The decision whether to use heritable human genome editing in a prospective child should, subject to principles I, II, and III, be left to the prospective parents.*
- *Principle V: Concerns about exacerbating social inequalities should be addressed by measures to increase access.*

These principles were embodied in suggested regulatory guidelines have been submitted for possible inclusion in the revised National Health Research Ethics Guidelines which set out the norms and standards for research in South Africa.

## Concluding Comments

Human genome editing presents the possibility of curing serious illness, as well as removing serious illness from an individual’s family tree. If it remains unregulated, there will remain a tension between the scientific community which wants to develop its research into marketable therapeutics and health needs of the broader population. Properly regulated, the risk of unethical research will be reduced and a rights-based approach to science will ensure that scientific progress may continue within legal parameters, and the legal rights of clinical trial participants as well as patients are protected. It is hoped that the suggestions submitted in respect of the South African national ethics guidelines will be well received and that South African developments may bring us closer to a clinical pathway to heritable human genome editing.
